# Local Transmission of Dengue in South Florida: A Case Report

**DOI:** 10.7759/cureus.65375

**Published:** 2024-07-25

**Authors:** Lizis O Rodriguez, Eli B Levitt, Nahal Khamisani, Sarah Nickle, Guillermo Izquierdo-Pretel

**Affiliations:** 1 Internal Medicine, Florida International University, Herbert Wertheim College of Medicine, Miami, USA; 2 Translational Medicine, Florida International University, Herbert Wertheim College of Medicine, Miami, USA

**Keywords:** dengue-related hospitalization, infectious diseases epidemiology, dengue thrombocytopenia, adult internal medicine, dengue virus infection

## Abstract

Dengue is a significant global health concern, primarily prevalent in tropical and subtropical regions, affecting approximately 400 million people annually worldwide. This case report highlights the emergence of dengue in South Florida and raises concerns about the possibility of a local transmission. Unlike most cases, the patients discussed had not recently traveled, prompting the need for further investigation into the local transmission of dengue. In October 2023, two adult brothers presented to the Emergency Department (ED) with symptoms including fever, chills, nausea, vomiting, rash, severe headache, and syncope. Laboratory tests revealed thrombocytopenia, atypical lymphocytes, and elevated liver enzymes. With this clinical picture, dengue was suspected, and management was initiated accordingly. Both patients denied recent travel and had no known dengue infections in the past but had been exposed to mosquitoes a week prior. Both patients were managed with supportive treatment and platelet transfusions, which led to clinical improvement. However, the laboratory tests for dengue diagnosis had to be sent to a third-party laboratory, resulting in delayed confirmation of the disease. We received confirmation of a positive dengue serology approximately a week after the first patient was discharged. Two suspected cases of dengue virus were confirmed. The availability of local laboratory tests is crucial for the early diagnosis and management of dengue. The study calls for increased awareness of the disease's severity and its risk factors, emphasizing early recognition, judicious use of intravenous fluids, and the need for accessible local diagnostic resources to facilitate timely patient care.

## Introduction

Dengue, a vector-borne viral disease transmitted by *Aedes aegypti* and *Aedes albopictus* mosquitoes, remains a formidable global health challenge. With an annual impact affecting approximately 400 million people, primarily in tropical and subtropical regions, dengue has been a recurrent public health concern, leading to substantial morbidity and mortality worldwide [1]. The clinical presentation of dengue is diverse, encompassing a spectrum of symptoms such as fever, myalgias, thrombocytopenia, and even bradycardia. Typically associated with travel to endemic regions like Africa and Caribbean countries such as Cuba and Puerto Rico, this disease has garnered attention due to its potential for local transmission, even in regions previously considered non-endemic [2]. As dengue is continuously expanding away from its endemic habitats, partly due to climate change, local transmission of dengue in the United States is expected to exponentially increase in the years to come [3]. In a letter sent to the editor of the New England Journal of Medicine (NEJM), it states that there were 18 locally acquired cases of dengue in 2019 in Florida [4]. Additionally, the Florida Arbovirus Surveillance Report noted that there were 176 locally acquired dengue cases in Florida in 2023 [5], indicating an increase in locally acquired cases. Knowledge of these cases should alter clinical care and promote better health outcomes.

This case report focuses on two individuals from South Florida who presented with dengue virus without a recent history of travel, bringing to light the potential for local outbreaks. With a delay in confirming the diagnosis due to limited local laboratory resources, this report underscores the need for accessible and rapid diagnostic capabilities to aid clinicians in the early recognition and management of dengue, particularly in areas experiencing an upsurge in local transmission. This unique presentation prompts further exploration into the factors influencing the emergence of dengue in non-endemic regions and the implications it carries for public health efforts. We aim to shed light on the changing dynamics of dengue and the necessity for preparedness in regions where it was previously uncommon. We followed the case report (CARE) guidelines [6].

## Case presentation

In the autumn of 2023, a 53-year-old man who was otherwise healthy presented to the Emergency Department (ED) of a large academic medical center in Miami with four days of acute onset of fever, chills, nausea, vomiting, abdominal pain, severe headache, and a sore throat, as well as two reported episodes of syncope and one episode of dark stool. The patient stated that he felt weak, and he found it difficult to sustain himself standing without support or assistance. He denied chest pain and shortness of breath. He denied any recent travel in the last three months. He last traveled six months prior to Cuba. His past medical history was unremarkable, and he reported not taking any medications. Labs were notable for thrombocytopenia, acute liver injury (high aspartate aminotransferase, alanine aminotransferase, and alkaline phosphatase), and the presence of atypical lymphocytes (Table 1). He was admitted for further workup with an initial diagnosis of a viral illness.

**Table 1:** Lab values at admission

Description	Patient 1	Patient 2	Reference Values
White cells	4.0	6.5	4.0–10.5 x 10^3^/mcL
Hemoglobin	16.0	15.5	13.3–16.3 g/dL
Platelets	28,000	100,000	140,000–400,000/mcL
Aspartate aminotransferase	341	41	15–46 unit/L
Alkaline phosphatase	72	49	38–126 unit/L
Total bilirubin	1.1	0.9	0.2–1.3 mg/dL

**Figure 1:**
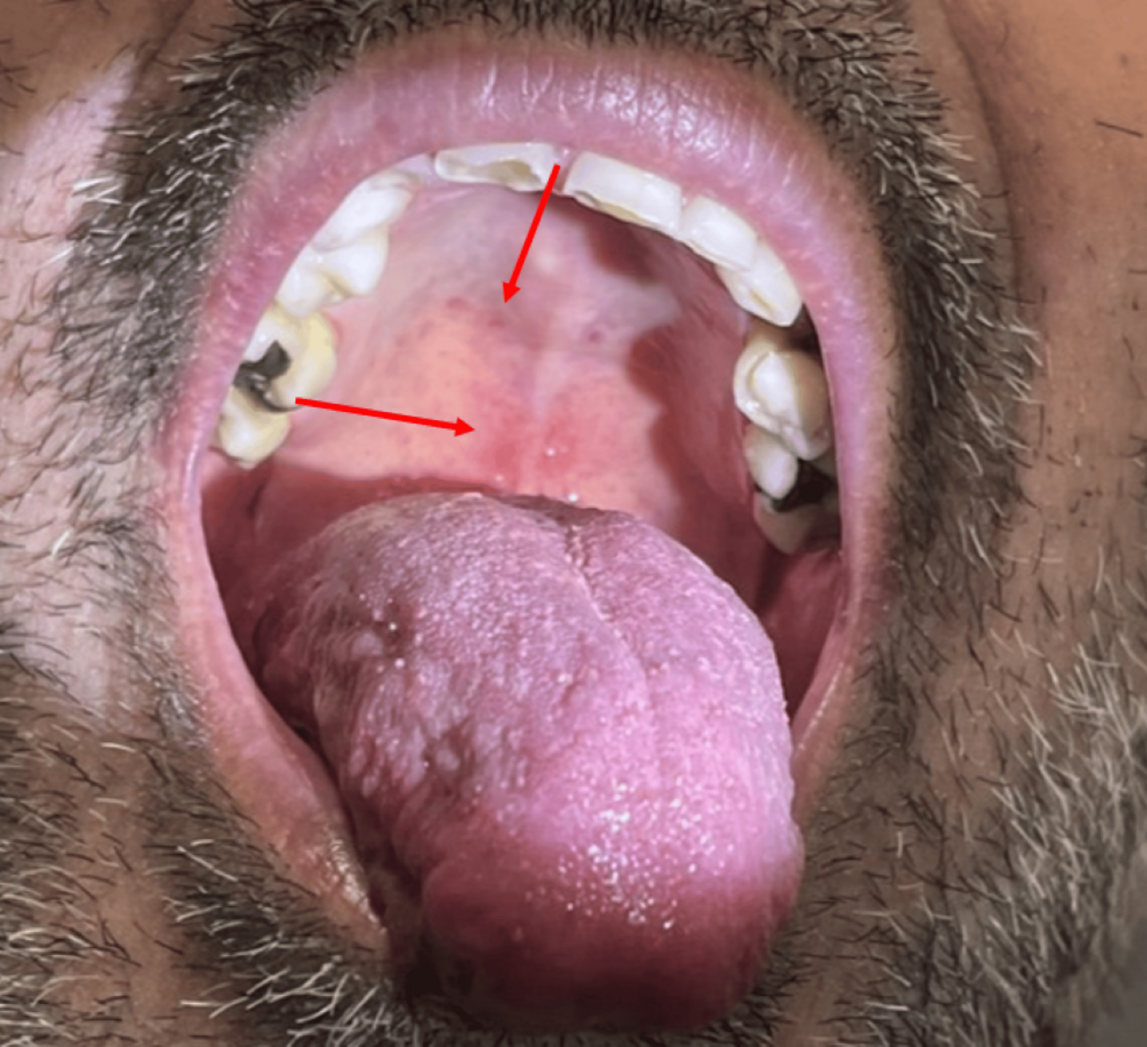
The inside of Patient 1's mouth The red arrows point to multiple areas of petechiae found on the patient's hard palate.

**Figure 2:**
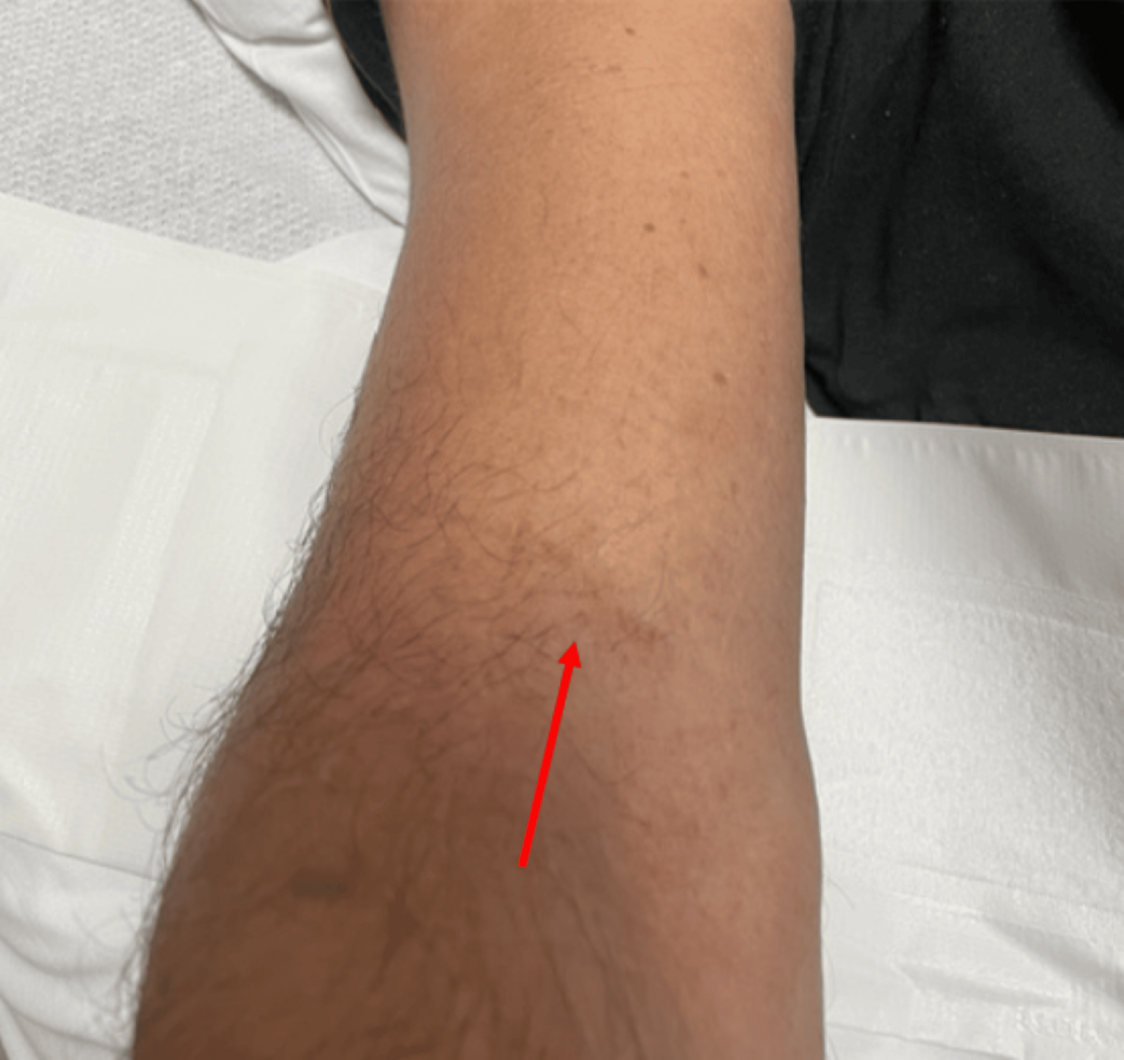
Linear petechiae found on Patient 1’s right forearm The red arrow shows residual petechiae that were formed after the compression of a blood pressure cuff on the patient's forearm.

**Figure 3:**
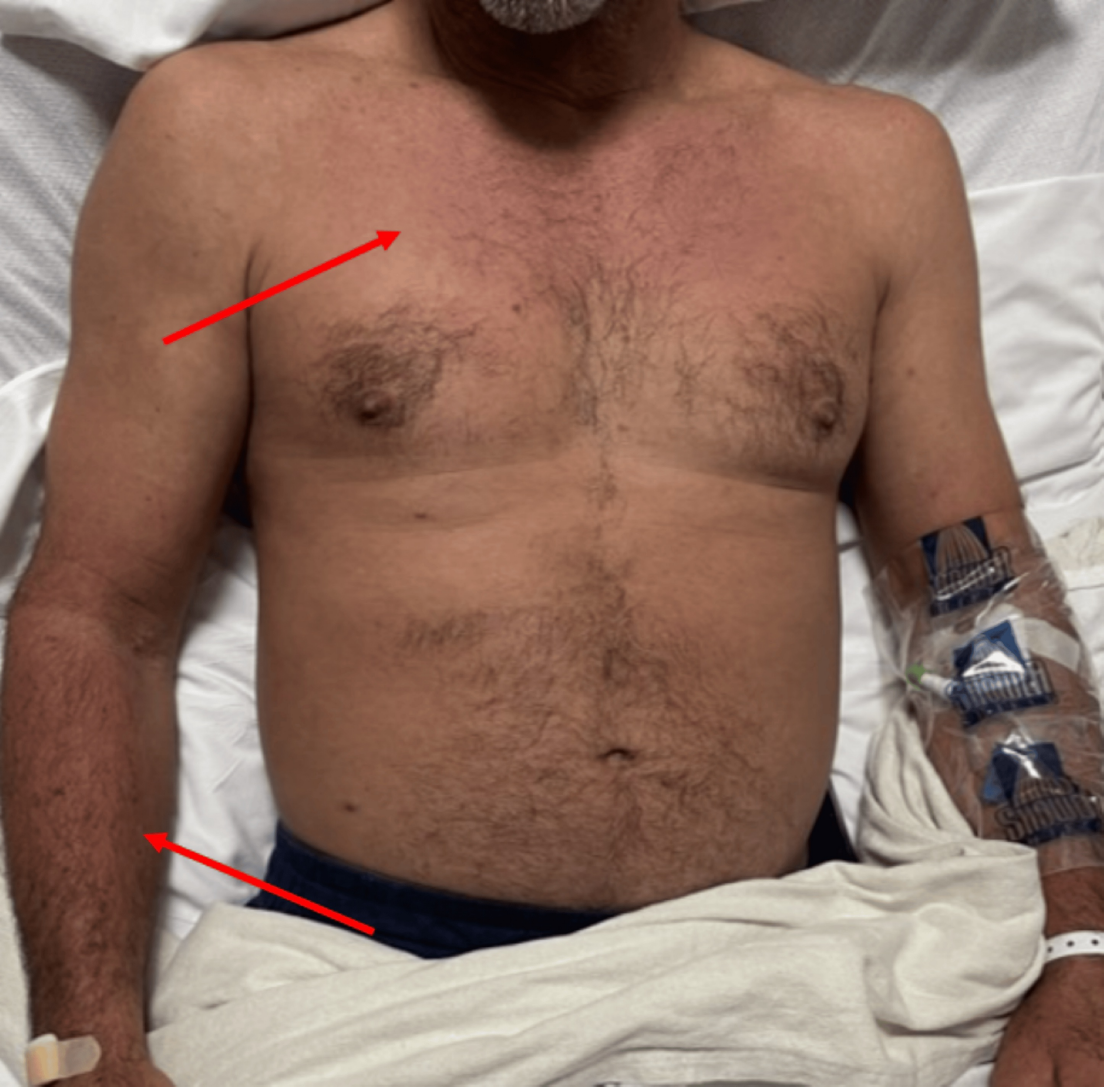
Rash on the chest, abdomen, and arms of Patient 1 The red arrows show a diffuse erythematous rash seen on the patient's chest and forearm.

**Figure 4:**
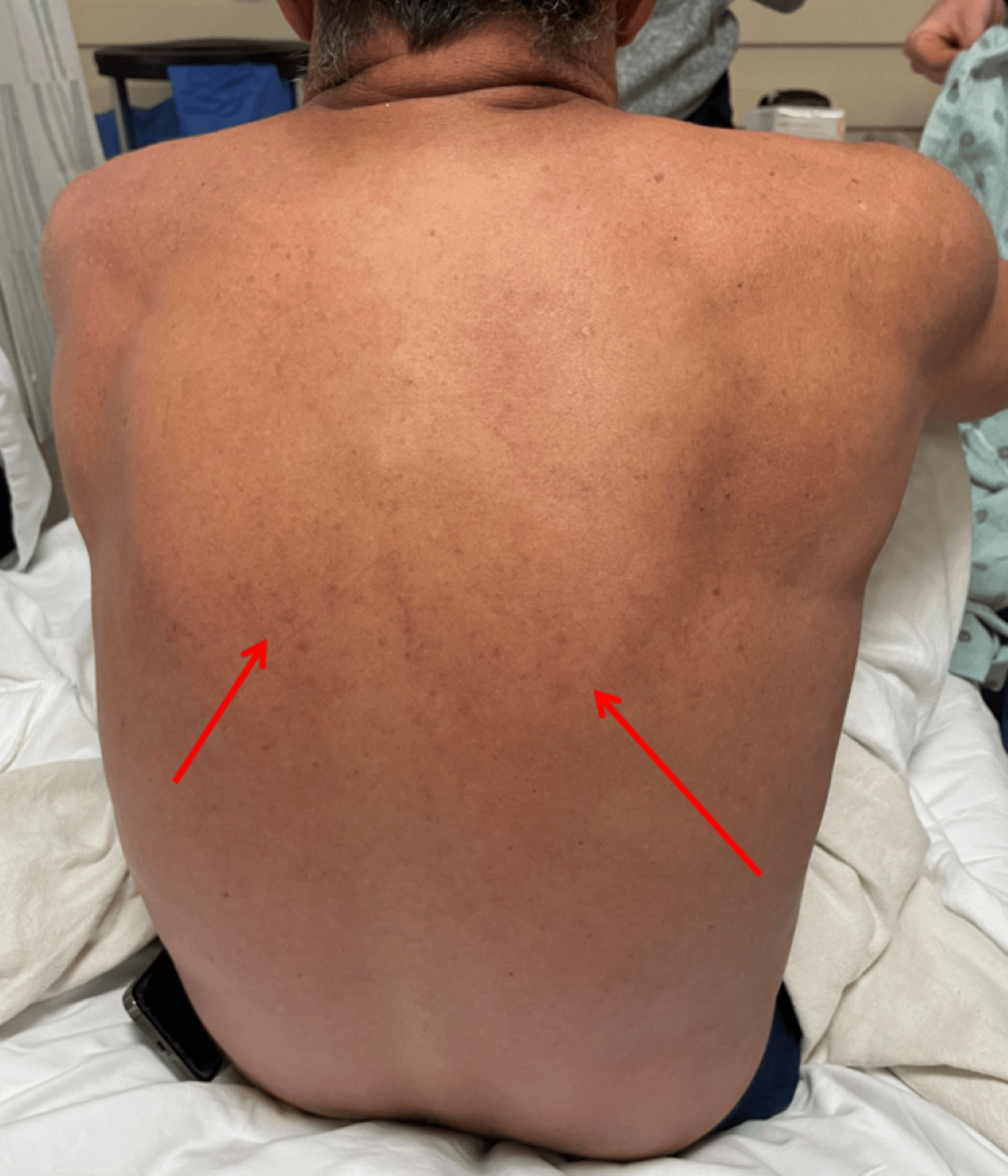
Diffuse rash found on Patient 1’s back The red arrows show a diffuse erythematous rash found on the majority of the patient's back.

Upon physical examination, the patient had palatal petechia (Figure 1) and right upper arm petechiae (Figure 2), where a hospital blood pressure cuff was used for routine measurement of blood pressure. This physical exam finding correlates with the tourniquet test. Additionally, he had a dissipating erythematous rash seen on his chest and arms (Figure 3) that extended to the entirety of his back (Figure 4). The abdominal examination revealed splenomegaly. The patient was noted to have orthostatic hypotension with a drop in systolic blood pressure of 20 mmHg.

**Figure 5:**
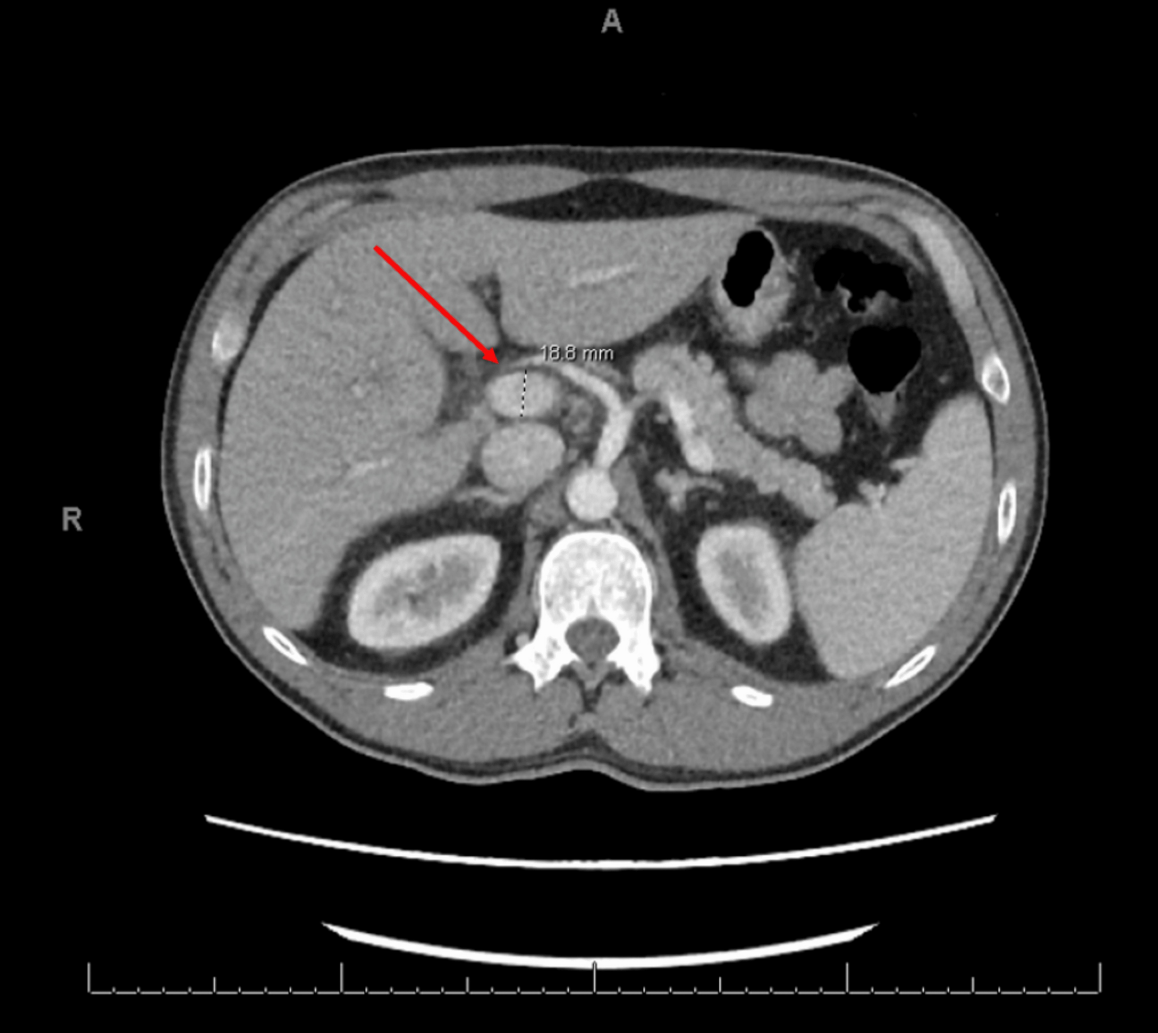
CT scan of the abdomen showing a dilated portal vein The red arrow demonstrates the dilated portal vein, measuring 18.8 mm.

**Figure 6:**
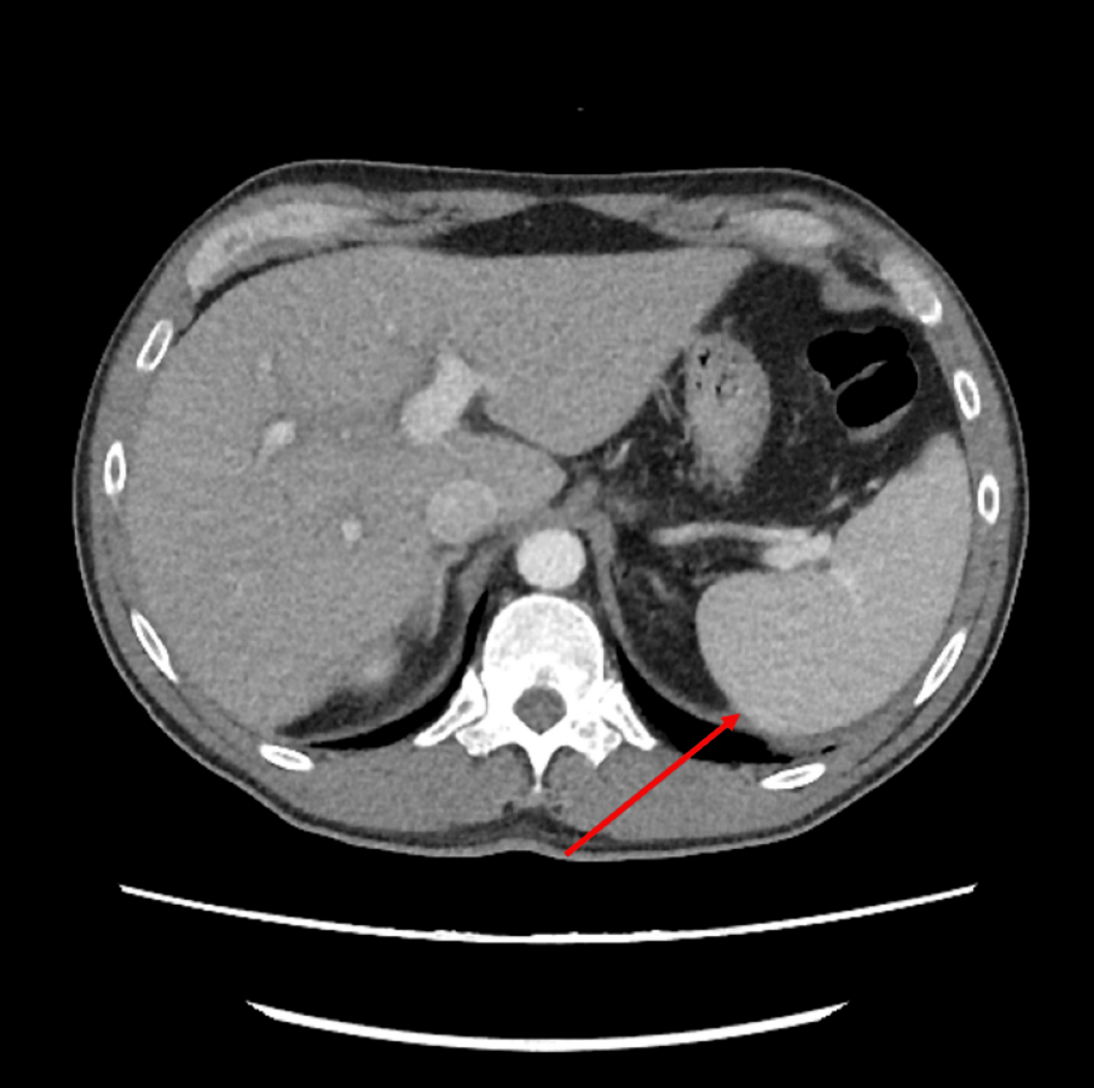
CT scan of the abdomen showing splenomegaly The red arrow demonstrates the patient's enlarged spleen.

A computed tomography (CT) scan of the abdomen obtained on the first day of hospitalization showed a dilated main portal vein (Figure 5), periportal edema, and splenomegaly (Figure 6) with no definitive evidence of cirrhosis. Upon initial evaluation, the primary care team hypothesized the possibility of dengue as the main diagnosis due to the thrombocytopenia and the clinical picture. Samples were collected and sent for the analysis of dengue immunoglobulin G (IgG), immunoglobulin M (IgM), and polymerase chain reaction (PCR) 1 or 3 ribonucleic acid (RNA) (positive seven days after collection). Additional testing for Chikungunya IgG, IgM, and PCR, Strep pharyngitis oral swab, hepatitis panel, Epstein-Barr virus (EBV), rheumatology panel, influenza A/B, respiratory-syncytial virus (RSV), severe acute respiratory syndrome coronavirus 2 (SARS-CoV-2), and human immunodeficiency virus (HIV) yielded negative results. Labs were drawn every 12 hours for blood cell differential counts and liver function monitoring.

The patient was treated with supportive treatment initially and intravenous fluids with normal saline (0.9%) for fluid resuscitation. No antibiotics were administered. On hospital day two, the patient's platelets dropped to 15,000/mcL (Table 2), and two platelet transfusions were started. After an infusion of 5 L of 0.9% normal saline and a platelet transfusion, the patient reported significant improvement in symptoms on hospital day three. The diffuse rash started to resolve, the hard palate showed less petechiae, and the patient reported less malaise. On his fourth day of admission, he reported that his 51-year-old brother was having similar symptoms as him and was on his way to the ED. Upon admission, the brother reported low-grade fevers, headache, and malaise. There was no erythematous rash observed at the time. The patient was admitted for further testing and treatment. Lab results showed isolated thrombocytopenia with a platelet count of 100,000/mcL (Table 2) initially. Each day of admission, the patient's platelets continued to drop. On day four, platelets were 18,000/mcL, and on day five, they were 8,000/mcL (Table 2). The patient was transfused with two units of platelets. Both patients had denied any recent travel or any close family members who traveled, no sick contacts, and no prior dengue infections. They stated that they were together a week prior to the first hospitalization, cleaning a shed with moldy, water-filled boxes that contained mosquito nests. They both reported that they were bitten by the mosquitoes while in the shed.

**Table 2:** Platelet count during the hospital course N/A: not applicable

Day	Patient 1	Patient 2
Day 1	28,000 (Low)	100,000 (Low)
Day 2	15,000 (Low)	58,000 (Low)
Day 3	24,000 (Low)	33,000 (Low)
Day 4	43,000 (Low)	18,000 (Low)
Day 5	73,000 (Low)	8,000 (Low)
Day 6	N/A	14,000 (Low)
Day 7	N/A	25,000 (Low)
Day 8	N/A	75,000 (Low)
Reference values (units): normal values range from 140,000 to 400,000/mcL		

The lab findings for both patients were delayed by approximately one week since the local hospital lab did not have the necessary tests for dengue IgG, IgM, and PCR and the necessity of sending laboratory tests to a third-party laboratory. The results for Patient 1 were sent on October 3, 2023, and returned on October 11, 2023, with a positive result for dengue virus 1 or 3 RNA. Table 3 shows the results of antibody testing for Patient 1, which includes a dengue IgG of 1.78 (positive) and 2.02 (positive) and dengue IgM of 4.60 (positive) and 3.38 (positive) on two distinct blood draws. The lab results for Patient 2 came back that same week (ordered on October 4, 2023, and verified on October 8, 2023), also with a positive result for dengue virus 1 or 3 RNA. Dengue virus was confirmed as the main diagnosis. Both cases have been reported to the Florida Department of Health.

**Table 3:** Antibody serology for dengue sent to a third-party laboratory

Patient, Day	Dengue IgG Antibody	Dengue IgM Antibody
Patient 1, Day 2	1.78 (Positive)	4.60 (Positive)
Patient 1, Day 3	2.02 (Positive)	3.38 (Positive)
Patient 2, Day 1	0.80 (Positive)	1.14 (Equivocal)
Patient 2, Day 2	1.21 (Positive)	1.49 (Equivocal)
Reference Values (interpretation)	< 0.80 (Negative), 0.80–1.09 (Equivocal), ≥ 1.10 (Positive)	≤ 1.65 (Negative), 1.66–2.83 (Equivocal), ≥ 2.84 (Positive)

## Discussion

The strengths of this case report include the importance of an increasing incidence of local dengue in South Florida, particularly in Miami, where local transmission has not traditionally been common. These two cases underscore the evolving nature of dengue, highlighting the potential for outbreaks in non-endemic areas. It is important for the academic and medical communities to spread awareness of the severity of this disease, its risk factors, and a timely clinical diagnosis. Certain clinical signs, such as abdominal pain, persistent vomiting, and tender hepatomegaly, should be recognized as ominous indicators of patient deterioration [7]. One retrospective study was performed on 130 diagnosed patients with dengue, where fever, myalgia, and headache were the most common symptoms [8]. Severe cases may result in plasma leakage and bleeding, ultimately leading to lethal outcomes [9]. Early clinical detection and prompt supportive treatment are essential aspects of management. Knowing how to test for the dengue virus is also an important part of confirming the diagnosis. It can be detected via laboratory tests in two ways: detection of the virus in the blood via PCR or detection of anti-dengue antibodies IgG or IgM in plasma, which are most sensitive during the febrile period of the disease [10]. In our case, these results were only available after the diagnosis, management, and discharge of these patients, highlighting the call for increased awareness of the clinical presentation and the possible benefits of rapid diagnostic testing.

The Centers for Disease Control (CDC) and various organizations are actively involved in public health measures to mitigate the risks of dengue on a large scale. Implementation of control programs in areas where dengue has been known to cohabitate can be a targeted area of improvement [11]. Some control measures happening worldwide include a vaccine for use in children aged 9-16 years with laboratory-confirmed previous dengue virus infection and living in areas where dengue is endemic. The vaccines have limited efficacy in preventing infection with all four serotypes [12], but new tetravalent vaccines are actively studied in places where dengue is endemic [13]. Another approach being taken by multiple countries includes the introduction of modified mosquitoes with the bacterium *Wolbachia*. Mosquitoes infected with such a bacterium do not have the potential to spread the dengue virus. Such measures have started to take effect in countries including Brazil, Indonesia, Colombia, and Singapore [12]. The availability of these tools to control dengue is important, but the spread of the disease is intertwined with the dramatic alterations brought by climate change [12]. It is hypothesized that the increase in temperature and rainfall brought upon by climate change has favored the increased spread of vector-borne diseases such as dengue [12]. Continued research and commitment to innovative approaches to controlling dengue are needed.

In response to a fatal case of dengue in 2021 at our institution, one of our local care teams worked with the CDC and the Florida Department of Health (FDOH) to raise awareness about the delayed diagnostic testing available for dengue [4]. At that time, the patient underwent laparoscopic surgery for acute cholecystitis, and complications included hemorrhage, multiorgan failure, and subsequent death. The patient had recently traveled to Cuba, and it was hypothesized that the infection was acquired abroad. Blood tests were drawn during her hospital stay, but positive results of the dengue serologic tests were reported postmortem [4].

The two patients in our report recovered after early detection and supportive care; however, diagnostic results of the serologic tests took eight days for Patient 1 and four days for Patient 2 to be reported. A potential change that could be implemented is to increase the availability of local laboratory tests in places where dengue transmission has become more prevalent. Local diagnostic tests may provide real-time results of possible dengue cases in cities where the virus is on the uprise, such as Miami, Florida. Early detection with clinical evaluation was essential in preventing life-threatening effects from occurring in these two patients, as our hospital had to send out the diagnostic tests to third-party labs. In our case, the estimated turnaround time for dengue serology results was seven days.

Referencing the coronavirus 2019 (COVID-19) pandemic, in April 2020, the National Institute of Health (NIH) launched the Rapid Acceleration of Diagnostics (RADx) Initiative, which aimed to provide fast and accurate tests nationwide to hospitals, laboratories, and the public [14]. It was determined that the availability of tests was a substantial component of controlling the spread of SARS-CoV-2 by creating awareness among those who were infected based on the risk of disease transmission [14]. A similar study by Zhang et al. highlighted the role that testing has on the behavior of those who were infected, reporting that those who had available testing were less likely to engage in behaviors that could potentiate the spread of the disease [15]. Unlike COVID-19, dengue is spread through a vector, but knowing the important role that available testing played during the COVID-19 pandemic has geared us to better inform the public on what health measures to take. Even for a vector-borne disease such as dengue, earlier access to diagnostic testing can help us educate the public on how best to prevent the spread of the disease during an outbreak.

In our case report, although the patients received adequate treatment with platelet transfusions and supportive care, the laboratory tests to confirm the diagnosis of dengue required follow-up with a third-party laboratory. This resulted in a confirmed diagnosis only after the patients were discharged. The broader implications of diagnostic testing will facilitate early detection and treatment of individuals who might show symptoms of dengue fever. This case report demonstrates the pressing need for accessible diagnostic tools and efficient control programs, underscoring the implications for public health preparedness in regions where the dengue virus has been on the rise. Local transmission of dengue in Miami-Dade, Florida, has been reported to have increased in 2022-2023, with a peak at 154 cases in 2023 as per the CDC’s National Arbovirus Surveillance System [16]. We recommend increasing readily available laboratory tests in local hospitals as areas with risk of dengue change. Early detection and treatment are key to a rapid recovery and prevention of severe outcomes.

In this case report, the dengue IgM antibody was only positive for Patient 1, whereas the dengue PCR 1 or 3 RNA was reported to be positive for both patients in this report. Despite this discrepancy, PCR testing can be used as early as the first day of the disease due to its high sensitivity, whereas IgM serology will turn positive after the fourth day of the disease [10]. Additionally, IgM serology has been deemed not to be fully reliable due to cross-reactivity with other flaviviruses [10]. Viral RNA can be isolated using reverse transcriptase real-time PCR or conventional PCR during the first one to seven days of the disease, mainly during the period of fever [10]. Ultimately, we conclude that the use of PCR with viral RNA as a serologic test should be the preferred method to confirm the dengue virus and avoid false negatives that may delay the diagnosis. In this case report, the positive PCRs support our diagnosis of dengue in both patients.

## Conclusions

The emergence of dengue in a region such as South Florida, where local transmission was not previously common, emphasizes the evolving nature of this disease. The delayed diagnostic results of these cases due to limited local laboratory resources highlight the potential importance of accessible and rapid diagnostic capabilities for dengue. This report emphasizes the necessity for heightened awareness among healthcare professionals regarding the potential for local outbreaks, even in non-endemic areas, and the need for early recognition and management. As dengue dynamics change and its reach extends to new regions, early detection and intervention remain pivotal in preventing severe outcomes. The findings also underscore the broader implications for public health preparedness in regions where dengue was once considered uncommon, urging the establishment of efficient control programs, appropriate clinical identification, and timely access to diagnostic tools to mitigate the impact of this disease.
